# Supplementary material for: Relationship between Aggressiveness, Self-Confidence, and Perceived Coach Support and Head Impact Exposure in Youth Football
Source: Sports (Basel). 2022 Jul 29;10(8):115. doi: 10.3390/sports10080115 (PMC9412463; doi:10.3390/sports10080115)
Supplement: Supplementary file 1 [file sports-10-00115-s001.zip › sports-1789893-supplementary.pdf]

Name:_________

Please read the instructions carefully before responding to the statements.

Think about your confidence and how your performance may affect your confidence ***generally.***

The statements below describe how you may feel ***generally*** about your confidence, answer each statement by circling the number that corresponds to how strongly you agree or disagree **generally**. Please try and respond to each item separately.

The terms ***competition*** refers to matches, tournaments or other competitive events.

Please answer the items as honestly and accurately as possible there are no right or wrong answers. Your response will be kept confidential.

	Strongly Disagree ⟵ Neutral ⟶ Strongly Agree
1. A bad result in competition, or game, has a very negative effect on my self-confidence.	1 2 3 4 5 6 7 8 9
2. My self-confidence goes up and down a lot.	1 2 3 4 5 6 7 8 9
3. Negative feedback from others does not affect my level of self-confidence.	1 2 3 4 5 6 7 8 9
4. If I perform poorly, my confidence is not badly affected.	1 2 3 4 5 6 7 8 9
5. My self-confidence is stable; it does not vary very much at all.	1 2 3 4 5 6 7 8 9
6. My self-confidence is not greatly affected by the outcome of competition.	1 2 3 4 5 6 7 8 9
7. If I make a mistake it hurts my self-confidence a lot.	1 2 3 4 5 6 7 8 9
8. My self-confidence remains the same, even if my athletic ability changes.	1 2 3 4 5 6 7 8 9

Name:_________

This questionnaire contains items that are related to your experience with your coach (trainer). Coaches have different styles in dealing with athletes, and we would like to know more about how you have felt about your encounters with your coach. Your responses are confidential. Please be honest and candid.

	Strongly Disagree			Neutral			Strongly Agree
1. I feel that my coach provides me choices and options.	1	2	3	4	5	6	7
2. I feel understood by my coach.	1	2	3	4	5	6	7
3. I am able to be open with my coach while engaged in athletics.	1	2	3	4	5	6	7
4. My coach conveyed confidence in my ability to do well at athletics.	1	2	3	4	5	6	7
5. I feel that my coach accepts me.	1	2	3	4	5	6	7
6. My coach made sure I really understood the goals of my athletic involvement and what I need to do.	1	2	3	4	5	6	7
7. My coach encouraged me to ask questions.	1	2	3	4	5	6	7
8. I feel a lot of trust in my coach.	1	2	3	4	5	6	7
9. My coach answers my questions fully and carefully.	1	2	3	4	5	6	7
10. My coach listens to how I would like to do things.	1	2	3	4	5	6	7
11. My coach handles people's emotions very well.	1	2	3	4	5	6	7
12. I feel that my coach cares about me as a person.	1	2	3	4	5	6	7
13. I don't feel very good about the way my coach talks to me.	1	2	3	4	5	6	7
14. My coach tries to understand how I see things before suggesting a new way to do things.	1	2	3	4	5	6	7
15. I feel able to share my feelings with my coach.	1	2	3	4	5	6	7

Name:_________

Below is a list of statements about **your competitiveness when playing football**. We would like to know how often these things happen to you. There is no right or wrong answer. Your responses are confidential. Please be honest and candid.

	Almost Never				Almost Always
I become irritable if I am disadvantaged during a match	1	2	3	4	5
I feel bitter towards my opponent if I lose	1	2	3	4	5
I get mad when I lose points	1	2	3	4	5
I show my irritation when frustrated during a game	1	2	3	4	5
I find it difficult to control my temper during a match	1	2	3	4	5
Official's mistakes make me angry	1	2	3	4	5
Violent behavior, directed towards an opponent, is acceptable	1	2	3	4	5
It is acceptable to use illegal physical force to gain an advantage	1	2	3	4	5
I taunt my opponents to make them lose concentration	1	2	3	4	5
I use excessive force to gain an advantage	1	2	3	4	5
I verbally insult opponents to distract them	1	2	3	4	5
Opponents accept a certain degree of abuse	1	2	3	4	5
